# Hepatoprotective effect of *Thymus vulgaris* extract on sodium nitrite-induced changes in oxidative stress, antioxidant and inflammatory marker expression

**DOI:** 10.1038/s41598-021-85264-9

**Published:** 2021-03-11

**Authors:** Mohamed Mohamed Soliman, Adil Aldhahrani, Mohammed M. M. Metwally

**Affiliations:** 1 Clinical Laboratory Sciences Department, Turabah University College, Taif University, P.O. Box 11099, Taif, 21944 Saudi Arabia; 2 Biochemistry Department, Faculty of Veterinary Medicine, Benha University, Benha, 13736 Egypt; 3 Department of Pathology, Faculty of Veterinary Medicine, Zagazig University, Zagazig, 44519 Egypt

**Keywords:** Cell biology, Molecular biology, Medical research

## Abstract

The herb thyme (*Thymus vulgaris*) has multiple therapeutic uses. In this study, we explored how *T. vulgaris* leaf extract protects liver cells against sodium nitrite-(NaNO_2_) induced oxidative stress. Mice were divided into four groups; each group received one of the following treatments orally: saline; *T. vulgaris* extract alone; NaNO_2_ alone; *or T. vulgaris* extract + NaNO_2_. Alanine aminotransferase (ALT), aspartate aminotransferase (AST), reduced glutathione (GSH), superoxide dismutase (SOD), malondialdehyde (MDA), IL-1β, IL-6, TNF-α, and total proteins were measured in serum using standard methods. TNF-α, hemooxygenase-1 (HO-1), thioredoxin, SOD, and GSH synthase, all of which are linked to oxidative stress, were measured using quantitative real-time PCR (qRT-PCR). In mice treated with *T. vulgaris* extract, the effect of NaNO_2_ on ALT and AST levels and total proteins was reduced, and its effect on antioxidant levels was reversed. Normally, NaNO_2_ causes hepatocyte congestion and severe hepatic central vein congestion. Tissues in the mice treated with *T. vulgaris* were restored to normal conditions. Our results demonstrate that NaNO_2_-induced hepatic injury is significantly reduced by pretreatment with *T. vulgaris* extract, which protects against hepatic oxidative stress and its associated genes at the biochemical, molecular, and cellular levels.

## Introduction

Nitrite is important to organ health. At very low concentrations, and within physiological levels, nitrite has antioxidant properties^1,2^. When reduced to nitric oxide (NO), which is critical for vascular homeostasis^3^, it plays a critical role in cell signaling, cellular immunity, and in the activation of certain regulatory proteins^3,4^. These regulatory proteins are vital to the maintenance of normal human health.

Human beings use nitrites in a wide variety of ways, which makes them hard to avoid. In industry, nitrite and its salts are used in preservatives, colorants, and in manufacturing rubber products and dyes; in food, nitrite is employed for its anti-microbial properties; and in medicine, it is used to treat cyanide poisoning, ischemic heart disease, and as a vasodilator^5–7^. Environmentally, improper waste disposal, over-reliance on nitrite-based fertilizers, and excessive use in food preservatives has led to chronic exposure and the accumulation of high nitrite-nitrate levels in humans.

Overexposure (due either to high concentrations or prolonged low-dose exposure) is toxic and can cause liver, kidney, or other organ failure^8^, methemoglobinemia^9^, and eventually death. Overexposure in humans occurs primarily through contaminated food or drinking water. After first affecting the GI tract, it passes into the circulation, where it reaches various other organs^10^, causing further oxidative stress and toxicity. It has been observed that oxidative stress is a key mediator of nitrite-induced oxidative damage^11^ and that treatment with antioxidants can ameliorate or even reverse this^12^. The possible mechanism by which NaNO_2_ decreases antioxidant enzyme activity is induction of reactive oxygen species (ROS), which mediate the oxidation of enzyme molecules or the binding of NO^13^.

In the liver, NaNO_2_ induced hepatic toxicity through inhibition of antioxidant activity and increasing the levels of pro-inflammatory cytokines^14^. Liver disease is often treated using synthetic chemical drugs, but these can cause serious undesirable side effects, including cirrhosis, cholestatic jaundice, and elevated serum transaminase levels. Consequently, there is a need to explore gentler and lower cost treatments for nitrite toxicity and liver disease. Many herbal compounds contain polyphenols and flavonoids, which have anti-inflammatory and anti-oxidative effects. This has led to increased pharmacological interest in certain herbal medications. Thyme (*Thymus vulgaris*), an herb commonly used in cooking throughout the world, has antiseptic, antimicrobial, carminative, and anti-oxidative properties^15,16^ and has been used to treat a range of human illnesses^17^.

In traditional medicine, *T. vulgaris* has been used in a variety of ways because of its antiseptic, antispasmodic, anti-asthmatic, bronchodilator, expectorant, antitussive, carminative, anthelmintic, anti-microbial, and antioxidant qualities. It has been used to treat dyspepsia, chronic gastritis, diarrhea, and tonsillitis^18^. *T. vulgaris* extracts are believed to contain important anti-inflammatory and immunomodulator factors^19^ with high free radical scavenging, anti-inflammatory, anti-platelet, anti-obesity, and anti-diabetic actions^17,20^.

Monoterpenes and their hydrocarbons, as well as phenolics such as rosmarinic acid and flavonoid derivatives^21^, are found in significant amounts in many species of thyme. The presence of these compounds renders thyme an important herb in research on antioxidants^22^. The aim of our study was to examine the protective effects of *T. vulgaris*, at both the cellular and biochemical levels, on NaNO_2_-induced hepatic oxidative stress.

## Materials and methods

### Chemicals and kits

Kits for liver biomarkers, aspartate transaminase (AST), and alanine transaminase (ALT) were purchased from *Clini Lab* (Almanial; Cairo, Egypt). Kits for reduced glutathione (GSH), malondialdehyde (MDA), superoxide dismutase (SOD), and total proteins were purchased from Bio-diagnostic (Giza, Egypt). Chemicals used, including NaNO_2_ and agarose, were purchased from Sigma-Aldrich (St. Louis, MO, USA). Kits for RNA reverse transcription were purchased from Fermentas (Thermo Fisher Scientific, Waltham, MA, USA). The QIAzol reagents were purchased from QIAGEN (Valencia, CA, USA).

### Preparation of *T. vulgaris* extract

*T. vulgaris* leaves were collected from Taif markets in April 2020. Plants were identified by Dr. Yassin Alsodani a botanist at the College of Science, Taif University. The protocol and the methods used in this study was complied with relevant Taif University, Saudi Arabia and international guidelines and legislations. The extract was prepared according to the method used by Abdel-Aziem et al.^23^; the leaves were first air-dried, then powdered. The powder (100 g) was immersed in 500 mL ethanol (70%) and mixed. After 24 h, the mixture was filtered through Whatman paper No.1, then evaporated at 44 ± 1 °C in a vacuum. The solid extract (with a yield of 14.5% per 100 g of original material) was freeze-dried and stored at -20 C°.

### Measurement of total polyphenolic and flavonoid compounds

Total polyphenols and flavonoids were estimated, with modifications, using a method previously described by Singleton et al.^24^, who originally evaluated the total polyphenolic compounds in licorice extract using Foline-Ciocalteu reagent. This method has since been further developed and refined^25,26^. We combined 200 μL of sample (in triplicate) with 1 mL Folin-Ciocalteu reagent (10% v/v) and 800 μL 7.5% (w/v) Na_2_CO_3_, which was then incubated for 30 min at 25 °C. The optical density was measured spectrophotometrically at 760 nm, calibrated using a gallic acid standard curve. Values are presented in terms of mg gallic acid equivalent per 1 g dry thyme extract. Total flavonoid content was determined using an aluminum chloride colorimetric method^27^, based on a rutin calibration curve (mg rutin equivalent per 1 g dry thyme extract).

### Gas chromatography–mass spectrometry (GC–MS)

GC–MS analysis was performed using a Focus GC model (Thermo Electron Corporation, Waltham, MA, USA) under specific conditions: (1) DB-5 capillary column (30 m × 0.32 mm, 0.50 mm); (2) column temperature 60 °C (1 min) to 180 °C at 3 °C/min; (3) injector and detector temperature 220 °C; (4) split ratio 1:10; (5) carrier gas He; flow rate: 1.0 mL/min. A sample aliquot of 1 μL was diluted in chloroform at a ratio of 1:10. GC retention indices and ratios were compared on polar columns with specific standard mass spectra^28^.

### Experimental design and animal handling

**Figure 1:**
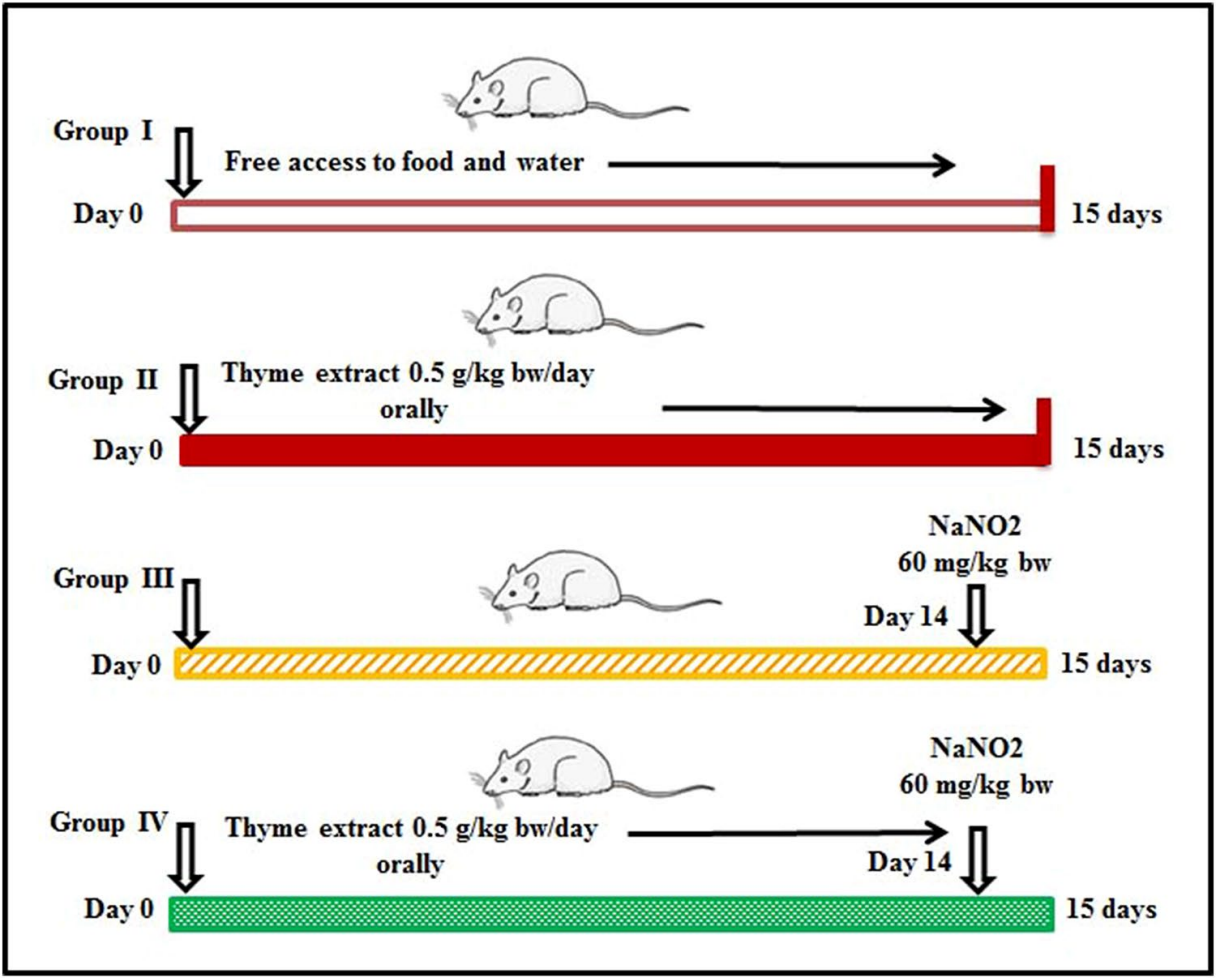
Schematic diagram showing the experimental design.

Twenty-eight mice (7 weeks old; 35 ± 2 g) were kept at 12 hr /12 hr light dark cycle in the animal quarters at Turabah University College, Taif University, Saudi Arabia. Mice were given free access to food and water and handled daily for 7 days to accustom them to human contact. The Ethical Committee of Turabah University College, Taif University approved all procedures and animal use for this study (project TURSP2020-09). Moreover, the study was carried out in compliance with the ARRIVE guidelines. The animals were split into four groups as follows: Group 1, the negative control group (CNT), received only saline orally; Group 2, the *T. vulgaris* control group, received 0.5 g/kg bw *T. vulgaris* extract orally for 15 days^23,29^; Group 3, the positive NaNO_2_ intoxication group, received 60 mg/kg bw NaNO_2_ orally on Day 14^30^; and Group 4, the protective group, received *T. vulgaris* extract as for Group 2, and then NaNO_2_ on Day 14, as for Group 3. Figure 1 shows the details of the experimental protocol. We chose the dose of 60 mg/kg bw of NaNO_2_ because, while this dose is not lethal, it will nevertheless cause significant toxicity, damaging tissues and cells, which can be measured clearly using biochemical assays^13,30,31^.

On Day 15, 24 h after the final treatment, the mice were anesthetized and then euthanized by decapitation. Liver samples were taken and preserved in Qiazol for RNA extraction and qRT-PCR; histological samples were prepared using Bouin’s solution; blood samples were collected and the extracted serum stored at -20 °C for later use.

### Biochemical assessments

Serum ALT and AST levels were measured using a colorimetric spectrophotometer following the procedures in the user’s manual. MDA levels were assessed using the technique developed by Ohkawa et al.^32^. SOD was measured using the method of Beutler et al.^33^, reduced GSH using the method of Nishikimi et al.^34^, and total protein levels were determined using the method of Lowry et al.^35^. These colorimetric methods were performed using a Bio-Rad Smart Tech Spectrophotometer according to the procedures in the product inserts provided with each kit. The reduced glutathione (GSH) and oxidized glutathione (GSSG) kits were purchased from Sigma-Aldrich Chemical (St. Louis, MO, USA). All chemicals used were of the highest purity and purchased from commercial suppliers in Saudi Arabia and Egypt.

### Cytokine measurements

Several different kits for measuring parameters in mice, including mouse IL-1β (cat. No. E-EL-M0037), IL-6 (E-EL-M0044), and TNF-α (E-EL-M0049) were purchased from Elabscience (Houston, TX, USA). Serum cytokine levels were determined using Sandwich-ELISA kits in accordance with the product inserts and reference tables available at the Clinical Laboratory Sciences Department, Turabah University College, Taif University.

### Quantitative real-time PCR (qRT-PCR) and gene expression

Total liver RNA was extracted and determined to be pure at 260/280 nm. Reverse transcription was performed using the Quanti-Tect reverse transcription kit with 1 µg total RNA, producing single-stranded complementary DNA (cDNA) using a two-step qRT-PCR reaction with a random primer hexamer. Amplification of cDNA was performed using SYBR Green master mix (Thermo Scientific, Waltham, MA, USA). Table 1 lists the primers used in thermal cycle qRT-PCR analysis, using the 2^–ΔΔCt^ method. β-actin was used as a ‘house-keeping’ (internal standard) gene, against which cDNA was normalized. Reactions were run on and analyzed using a 7500 FAST Real-Time PCR Detection System (Applied Biosystems, Foster City, CA, USA). qRT-PCR conditions were: 95 °C for 10 min (1st denaturation stage) and 40 cycles of 95 °C for 15 s (2nd denaturation stage) followed by 60 °C for 1 min (annealing and extension stage).

**Table 1:** Primers used for quantitative real time PCR in mice.

Gene	Accession number	Product size (bp)	Direction	Primer Sequence (5′–3′)
Thioredoxin	NM_011660.3	137 bp	Sense	5′-TGGAGAGGTGGCTCAGTCGT-3’
			Antisense	5′-GTGCCCATGGTGGCCAGTAG-3’
HO-1	NM_010442.2	126 bp	Sense	5′-CGCCTCCAGAGTTTCCGCAT-3’
			Antisense	5′-GACGCTCCATCACCGGACTG-3’
SOD	NM_011434.2	157 bp	Sense	5′-AGGACGGTGTGGCCAATGTG-3’
			Antisense	5′-CGGCTCCCAGCATTTCCAGT-3’
GSH synthase	NM_008180.2	139 bp	Sense	5′-CAGCCAGAACCCAGCCTTCC-3’
			Antisense	5′-GCGATTCAGGCCCAGGAACA
TNF-α	NM_013693.3	131 bp	Sense	5′-CACCATGAGCACAGAAAGCA-3’
			Antisense	5′-CTGCCACAAGCAGGAATGAG-3’
β-actin	NM_007393.5	140 bp	Sense	5′-CCAGCCTTCCTTCTTGGGTA-3’
			Antisense	5′-CAATGCCTGGGTACATGGTG-3’

Changes in intensity and expression of the specific genes listed in Table 1 under Analysis were determined using comparative cycle threshold (CT) values.

### Histological examinations

After decapitation, typical tissue specimens from the livers of all animals were harvested according to a standardized necropsy protocol^36^ and immediately fixed in Bouin's solution for 24 h. The fixed specimens were thoroughly washed under running tap water, dehydrated in an ascending series of ethanol concentrations (70%, 80%, 90%, and 100%; 2 h each), cleared in two changes of xylene (1 h each), impregnated in two changes of soft paraffin (2 h each), embedded in paraffin blocks, cut into Sects. 4 μm thick, and routinely stained with hematoxylin and eosin following standard processing and staining protocols^37^. The stained sections were examined microscopically and any histopathological alterations observed were recorded. This was followed by multiparametric quantitative lesion scoring for the hepatic parenchyma in all groups following the protocol described by Khalil et al.^38^ with a few modifications. For each animal, five randomly selected, non-duplicated microscopic profiles (40 × objective) were captured using an AmScope digital camera attached to an Olympus light microscope. These images were analyzed to allow calculation of the ratio of the area fractions of the hepatic central veins, portal blood vessels, sinusoids, necrosis, hemorrhage, fatty change, and leukocytic aggregations to the total areas using the image analysis software ImageJ (version 1.51v; Research Services Branch, NIH, Bethesda, MD, USA). The proportion of hepatic cells that exhibited pyknosis, single-cell necrosis, and vacuolar and hydropic degeneration with respect to the total number of hepatocytes per image was calculated subjectively. The frequency with which other lesions occurred (Kupffer cell hyperplasia, leukocytic infiltration, cholangiocyte hyperplasia and/or necrosis, and cholestasis) was determined by counting the number of lesions per image. Eventually, the results were expressed as percentages (mean ± SEM).

### Statistical analysis

Seven mice per group were selected and one-way ANOVA and Dunnett's post hoc descriptive tests were performed using SPSS software version 22 (SPSS, IBM, Chicago, IL, USA) for Windows. The data are presented as mean ± standard error of mean; statistical significance is p < 0.05, indicated by letters with different symbols**.

### Ethical Statement

All experimental procedures were carried out under National Institutes of Health Guidelines for the care and use of laboratory animals, and all procedures designed to minimize the suffering of animals were followed.

## Results

### Analysis and detection of *T. vulgaris* content

Analysis (using gallic acid as a standard curve) revealed that ethanolic *T. vulgaris* extract contained rosmarinic acid (38.23 mg/g dry weight thyme extract), luteolin-7-O-glucoside (17.3 mg/g), and caffeic acid (1.96 mg/g). These results are shown in Table 2. Concentrations of phenol and flavonoids were 221.65 and 115 mg/g, respectively, using rutin as a standard calibration curve (Table 1).

**Table 2:** Total Phenolic and flavonoids compounds identified in ethanolic extract of *Thymus vulgaris* for triplicate samples.

No	Name	Concentration
1	Caffeic acid	1.96 ± 0.53
2	Rosmarinic acid	38.23 ± 2.33
3	Luteolin	–
4	Luteolin-7-O-gluocoside	17.3 ± 1.034
5	Luteolin-7-O-rutinoside	–-
6	Apiginin	–
7	Apiginin–7-O-glucoside	–
8	Apiginin-7-O-rutinoside	–
9	Hesperdine	–
10	Hesperdine-7-O-glucoside	–
11	Hesperdine-7-O-rutinoside	–
12	Total Phenols	221.56 ± 6.8
13	Total flavonoids	115 ± 2

### GC–MS analysis

Table 3 shows the major compounds contained in *T. vulgaris* extract. Ten major compounds were detected in *T. vulgaris* extract following GC–MS analysis; the molecular weight (MW), retention time (RT), and peak area vary among these compounds. The hydroxyl (OH), carbonyl (CO), acetyl (COCH_3_), and nitro (NO_2_) functional groups are also shown in Table 3.

**Table 3:** Compounds identified by Gas Chromatography-Mass Spectrometry (GC.MS) in ethanolic extract of *Thymus vulgaris*.

Item	RT (min)	Peak Area %	Class name	Molecular weight	Molecular Formula
1	9.7	2.51	Pyrrole derivative chelated with Nickel	715	C_44_H_27_N_5_NiO_2_
2	11.58	2.54	17-(5-ethyl-6-methylheptan-2-yl)-10,13-dimethyl-2,3,4,7,8,9,10,11,12,13,14,15,16,17-tetradecahydro-1H-cyclopenta[a]phenanthren-3-ol	414	C_29_H_50_O
3	11.69	2.44	3-((tert-butyldimethylsilyl)oxy)-13-methyl-7,8,9,11,12,13,14,15,16,17-decahydro-6H-cyclopenta[a]phenanthrene-16,17-diyl bis(2,2,3,3,3-pentafluoropropanoate)	694	C_30_H_36_F_10_O_5_Si
4	27.99	2.9	10-hydroxy-3a,6,6,9a,11a-pentamethyl-1-(6-methylheptan-2-yl)hexadecahydrocyclopenta[7,8]phenanthro[8a,9-b]oxiren-7-yl acetate	502	C_32_H_54_O_4_
5	28.29	2.56	1-(2-hydroxy-7,9a,11b-trimethylhexadecahydrocyclopenta[1,2]phenanthro[8a,9-b]oxiren-9-yl)ethanone	346	C_22_H_34_O_3_
6	32.39	2.97	8-methoxy-10a,12a-dimethyl-5-methylene-1-(6-methylheptan-2- yl)hexadecahydrocyclobuta[m]cyclopenta[a]phenanthren-6(4H)-one	454	C_31_H_50_O_2_
7	41.69	2.44	N-(2-(2-acetyl-7-methoxy-1H-indol-3-yl)ethyl)benzenesulfonamide	372	C_19_H_20_N_2_O_4_S
8	44.62	2.69	dimethyl 3-(4-(5-hydroxy-1-(methoxycarbonyl)-1-methyldecahydronaphthalen-2-yl)butyl)-1-methoxy-7-methylnaphthalene-2,6-dicarboxylate	568	C_33_H_44_O_8_
9	46.61	2.96	Ethyl 7-(4-hydroxy-3-methoxy-5-nitrophenyl)-5-methyl-4,7-dihydro-[1,2,4]triazolo[1,5-a]pyrimidine-6-carboxylate	375	C_16_H_17_N_5_O_6_
10	50.18	3.16	5,5′,8,8′-tetrahydroxy-6,6′-dimethyl-[2,2′-binaphthalene]-1,1′,4,4′-tetraone	406	C_22_H_14_O_8_

### Serum hepatic biomarkers

We observed elevated ALT and AST levels and lower total proteins in the NaNO_2_-intoxicated groups (Groups 3 and 4), which indicated liver damage and dysfunction. In the group pretreated with *T.vulgaris* extract (Group 4), which received *T. vulgaris* extract for 14 days and then NaNO_2_ for 24 h, we observed less pronounced changes in ALT and AST levels and total proteins (Table 4). Pretreatment with *T. vulgaris* extract normalized and returned ALT and AST levels and total proteins to their relative control levels.

**Table 4:** Protective effects of *Thymus vulgaris* extract against NaNO_2_ induced alteration in liver biomarkers in mice.

	ALT (U/l)	AST (U/l)	Total proteins (mg/dl)
Control	17.3 ± 0.6	24.9 ± 0.9	81.6 ± 6.9
Thyme Extract	23.5 ± 5.1	20.4 ± 1.8*	78.3 ± 2.5
NaNO_2_	93.5 ± 4.3^#^	88.1 ± 3.3^#^	42.5 ± 4.5^#^
Thyme Extract + NaNO_2_	44.5 ± 1.5^$^	41.6 ± 4.7^$^	66.5 ± 4.8^$^

Values are means ± standard error (SEM) for 7 different mice per each treatment. Values are statistically significant at *p < 0.05 versus control; ^#^*p* < 0.05 versus control and thyme extract groups. ^$^*p* < 0.05 versus NaNO_2_. ALT: alanine transaminase; AST: aspartate transaminase.

### Impact of *T. vulgaris* extract on oxidative stress biomarkers

Increased levels of MDA (a biomarker of lipid peroxidation), along with lower SOD serum levels, indicated tissue damage in the NaNO_2_-intoxicated mice (Group 3). Group 2 mice, who received *T. vulgaris* only, exhibited moderate increases in SOD and GSH. In the NaNO_2_-intoxicated mice (Group 4) pretreated with *T. vulgaris* extract, we observed alleviated GSH and SOD levels and a decrease in MDA levels, providing evidence for the ameliorative effect of the *T. vulgaris* extract against NaNO_2_-induced hepatic toxicity (Table 5). The effect of *T. vulgaris* extract on the glutathione system was evaluated by determining the ratio of GSH to oxidized glutathione (GSSG) in serum from NaNO_2_-intoxicated mice (Table 5). The *T. vulgaris* extract had a positive effect on the glutathione status in NaNO_2_-injected mice by causing a concomitant increase in GSH levels and decrease in GSSG levels, which restored the GSH/GSSG ratio to control levels. The profound decrease in the GSH/GSSG ratio induced by NaNO_2_ was normalized by *T. vulgaris* treatment for 15 days.

**Table 5:** Protective effects of *Thymus vulgaris* extract against NaNO_2_ induced alterations in antioxidant activity in mice.

	MDA (nmol/ml)	SOD (U/ml)	GSH (nmol/l)	GSSG (nmol/l)	GSH/GSSGratio
Control	26.9 ± 0.89	3 ± 0.3	2.5 ± 0.6	1.1 ± 0.01	2.2 ± 0.02
Thyme Extract	28.3 ± 0.79	4.5 ± 0.7	3.3 ± 0.3	1.2 ± 0.01	2.73 ± 0.01
NaNO_2_	69.2 ± 5.5^#^	1.6 ± 0.3^#^	1.1 ± 0.01^#^	0.79 ± 0.01^#^	1.25 ± 0.02^#^
Thyme Extract + NaNO_2_	36.8 ± 1.4^$^	3.1 ± 0.6^$^	2.7 ± 0.2^$^	1.01 ± 0.02^$^	2.43 ± 0.07^$^

Values are means ± SEM for 7 different mice per each experiment. Values are statistically significant at ^#^*p* < 0.05 versus control and thyme extract groups. ^$^*p* < 0.05 versus NaNO_2_.

### Mitigated impact of *T. vulgaris* extract on inflammatory cytokines

To examine the destructive impact of NaNo_2_ on the immune state of mice, we measured the levels of pro-inflammatory cytokines. NaNO_2_ injection increased serum levels of IL-1β, IL-6, and TNF-α in the NaNO_2_-intoxicated mice (Groups 3 and 4). Pretreatment of NaNO_2_-intoxicated mice with *T. vulgaris* (Group 4) mitigated these effects (Table 6), which eventually returned to normal levels. *T. vulgaris* extract normalized all altered pro-inflammatory cytokines.

**Table 6:** Protective effects of *Thymus vulgaris* extract against NaNO_2_ induced changes in proinflammatory cytokines.

Measurements	Control	Thyme extract	NaNO_2_	Thyme extract + NaNO_2_
IL-1β (pg/ml)	156.7 ± 1.5	163 ± 24	308.2 ± 28^#^	165.7 ± 29.8^$^
IL-6 (pg/ml)	38.6 ± 1.4	43.7 ± 7	92.3 ± 3.8^#^	65.7 ± 13.3^$^
TNF-α (pg/ml)	235.3 ± 7.3	259 ± 19.3	427.3 ± 31.3^#^	273.7 ± 23^$^

Values are means ± SEM for 7 different mice per each experiment. Values are statistically significant at ^#^*p* < 0.05 versus Control and Thyme Extract groups. ^$^*p* < 0.05 versus NaNO_2_. NaNO_2_: Sodium nitrite.

### Ameliorative effect of *T. vulgaris* extract on quantitative expression of liver genes

**Figure 2:**
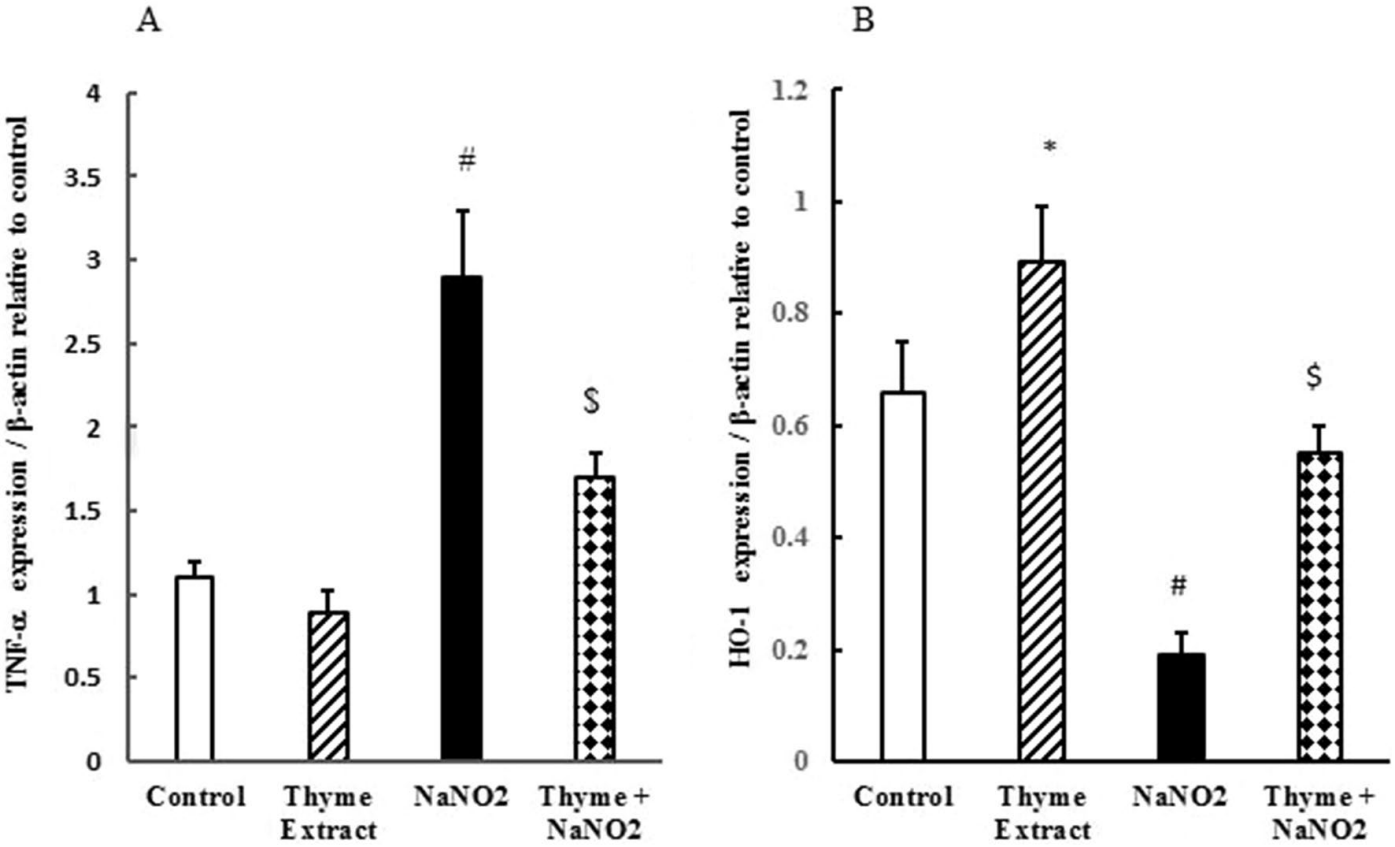
Ameliorative effect of *T. vulgaris* extract on mRNA expression of TNF-α (**A**) and HO-1 (**B**) in NaNO_2_-treated mice. Graphic presentation of liver mRNA expression by qRT-PCR analysis of TNF-α (**A**) and HO-1 (**B**) in different groups of mice relative to β-actin. ^*^p < *0.05* versus the control group; ^*#*^*p* < *0.05* versus the control and *T. vulgaris* extract groups; and ^*$*^*p* < *0.05* versus the NaNO_2_-treated group.

Nitrite toxicity induced liver damage and hepatic dysfunction as shown by the increases in hemeoxygenase-1 (HO-1), an oxidative stress biomarker, and TNF-α, an inflammatory cytokine (Fig. 2A,B). Gene expression for TNF-α mRNA was downregulated in the group pretreated with *T. vulgaris* extract (Fig. 2A). Conversely, the HO-1 gene was upregulated in the *T. vulgaris*-treated group (Fig. 2B). The ameliorative effects of *T. vulgaris* extract on both TNF-α and HO-1 expression and its potential to restore and recover gene alterations caused by NaNO_2_ intoxication are shown in Fig. 2A,B.

### Impact of *T. vulgaris* extract on quantitative expression of antioxidant genes in liver

**Figure 3:**
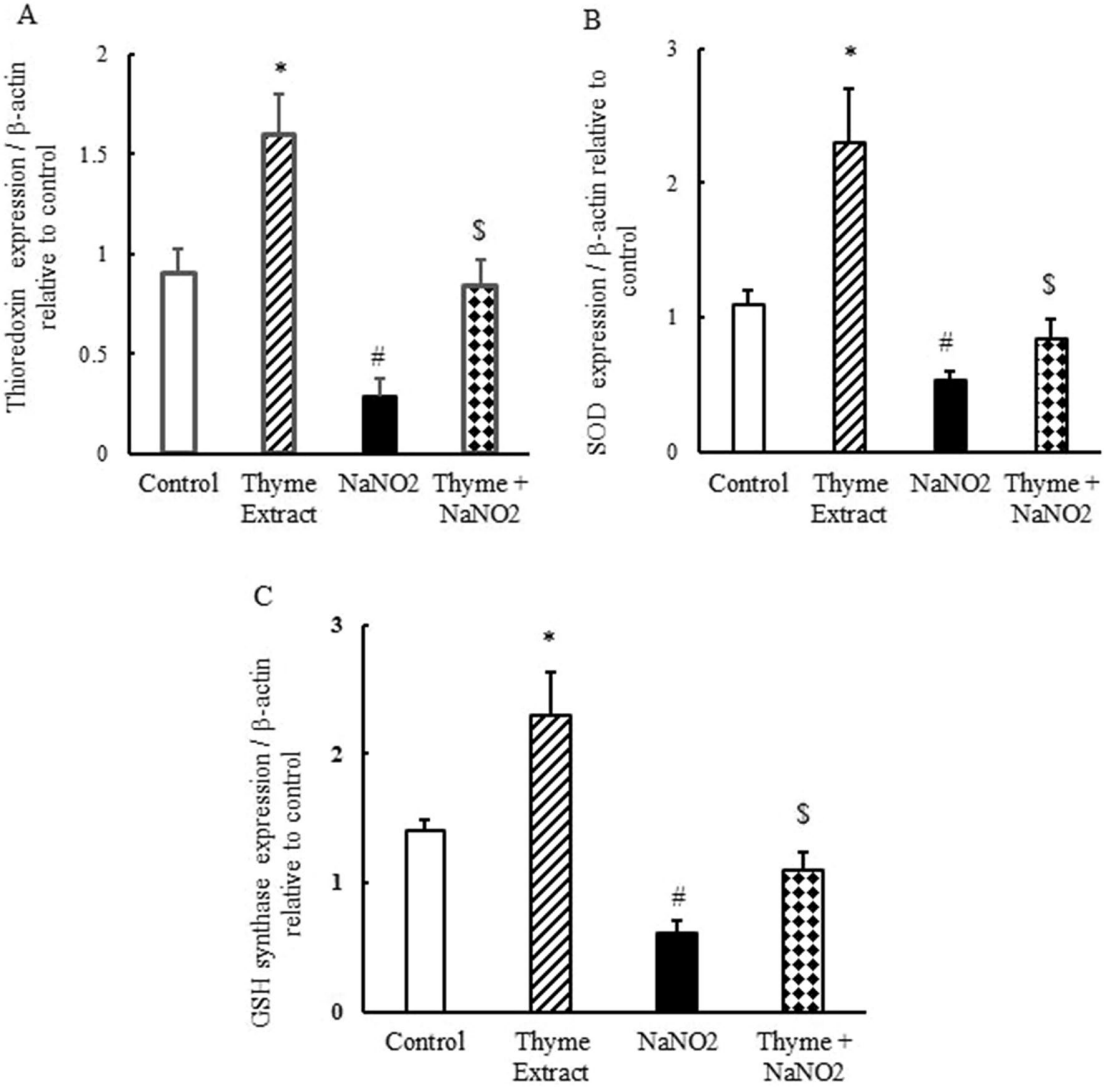
Ameliorative effect of *T. vulgaris* extract on mRNA expression of thioredoxin (**A**), SOD (**B**), and GSH synthase (**C**) in NaNO_2_-treated mice. Graphic presentation of liver mRNA expression by qRT-PCR analysis of thioredoxin, SOD, and GSH synthase in different groups of mice relative to β-actin. ^*^p < 0.05 versus the control group; ^*#*^*p* < 0.05 versus the control and *T. vulgaris* extract groups; and ^*$*^*p* < 0.05 versus the NaNO_2_-treated group.

qRT-PCR results showed that NaNO_2_ downregulated thioredoxin, SOD, and GSH synthase mRNA expression (Fig. 3A–C), leading to oxidative stress in the liver. The *T.vulgaris-*treated group (Group 2) showed positive correlation in the antioxidant genes examined, as *T. vulgaris* upregulated thioredoxin, SOD, and GSH synthase mRNA expression, demonstrating that the extract has important antioxidative properties (Fig. 3). In the group pretreated with *T. vulgaris* extract for 14 days followed by NaNO_2_ intoxication for 24 h (Group 4), the altered mRNA expression of the examined genes returned to within the normal range as a result of pretreatment.

### Histopathological examination

**Figure 4:**
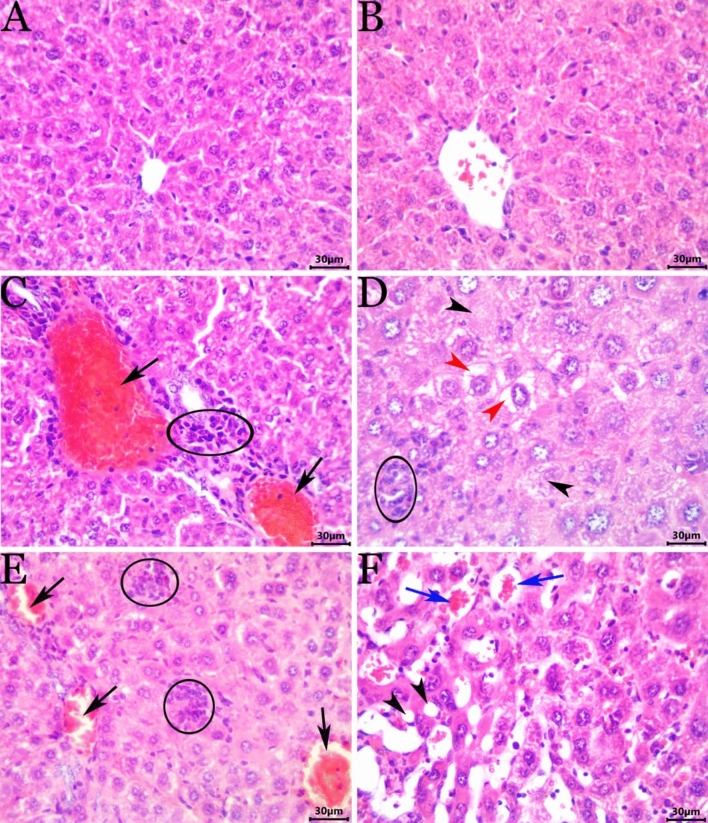
Representative photomicrograph of hematoxylin and eosin stained hepatic tissue sections showing the effects on the hepatic histology of exposure to NaNO_2_ and/or *T. vulgaris*. (**A**) and (**B**), normal histological photomicrographs in the control and *T. vulgaris*-treated mice, respectively. (**C**) and (**D**), livers of the NaNO_2_-treated mice showing portal congestion (arrows), inflammatory cell infiltrate (ellipses), cellular swelling associated with hydropic degeneration (red arrowheads), and single-cell necrosis (black arrowheads). (**E**) and (**F**), livers of NaNO_2_ + *T. vulgaris*-treated mice showing focal mononuclear cell aggregates (ellipses), fatty change (black arrowheads), congested blood vessels (black arrows), and sinusoids (blue arrows).

Upon microscopic examination of the specimens taken from the control and *T. vulgaris*-treated animals, we observed normal hepatic histological architectures: roughly hexagon-shaped hepatic lobules with sinusoids converging from the periphery to the central vein and portal canals present at approximately three of the six angles of the lobule. The hepatic parenchyma between the portal canals consisted of hepatocytes arranged in cell plates (Fig. 4A,B). Exposure to NaNO_2_ induced a wide variety of hepatopathic histological alterations, including circulatory changes (congestion of the central and portal veins and sinusoids), hepatocyte degeneration and necrosis (pyknosis, cellular swelling associated with vacuolations due to vacuolar and hydropic degenerations or fatty changes, and single cell necrosis), and inflammatory changes (intralobular and/or leukocytic infiltration or aggregations) (Fig. 4C,D). Interestingly, the biliary system did not show any noticeable histological alterations. *T. vulgaris* showed marked hepatoprotective effects against the NaNO_2_-induced hepatopathy. Although the hepatic tissue sections from the NaNO_2_ + *T. vulgaris*-treated animals were not completely histologically normal, we observed a significant reduction in the severity and frequency of the NaNO_2_-induced histological changes. The most frequent lesions in this group were vacuolar vascular congestion, sinusoidal dilatation, hepatocyte vacuolation, and tiny mononuclear cell aggregations (Fig. 4E,F). The overall hepatic lesion scoring changes induced by NaNO_2_ and possible amelioration by thymus extract are shown in Table 7.

**Table 7:** Analysis and lesion scoring in the hepatic tissues of mice in response to NaNO_2_ and *thymus vulgaris* treatment.

Lesion	Control	Thymus extract	NaNO_2_	GA3 + NaNO_2_
Central vein	2.2 ± 0.4^a^	2.17 ± 0.03^a^	2.7 ± 0.1^a^	2.4 ± 0.06^a^
Portal blood vessels	1.4 ± 0.06^a^	1.33 ± .0 07^a^	3.9 ± 0.13^b^	2.2 ± 0.09^c^
Sinusoidal spaces	6.3 ± 0.12^a^	6.7 ± 0.1^a^	9.01 ± 0.2^b^	8.0 ± 0.1^c^
fatty change	0^a^	0^a^	0.6 ± 0.1^b^	0.18 ± 0.09^c^
Focal leukocytic aggregation	0^a^	0^a^	0.77 ± 0.02^b^	0.29 ± 0.1^c^
Focal necrosis	0^a^	0^a^	0.1 ± 0.07^b^	0.04 ± 0.01^c^
Vacuolar and hydropic degeneration	0^a^	0^a^	26.2 ± 4.8^b^	17.5 ± 4.6^c^
Hepatocyte pyknosis	0^a^	0^a^	2.1 ± 0.4^b^	0.6 ± 0.2^c^
single-cell Necrosis	0^a^	0^a^	1.6 ± 0.4^b^	0.4 ± 0.1^c^
Cholestasis	0^a^	0^a^	0.0^a^	0. 17 ± 0.002^c^
cholangeocyte hyperplasia	0^a^	0^a^	0^a^	0^a^
Von Kupffer cell hyperplasia	0^a^	0^a^	4.6 ± 0.2^b^	3.2 ± 0.3^c^
Inflammatory infiltrate	0^a^	0^a^	23 ± 0.5^b^	9.1 ± 2.9^c^

Values are expressed as means ± SEM for 7 different mice per each treatment. Values with different letters means signficant at p < 0.05 beteween groups.

## Discussion

Our study confirmed that NaNO_2_ intoxication increases the levels of ALT and AST in serum, decreases total proteins, affects cytokine levels and gene expression (leading to an imbalance in antioxidant mechanisms), and causes histopathological changes, resulting in severe oxidative stress in liver tissues. However, pretreatment with *T. vulgaris* extract prevented or reversed these effects, confirming the antioxidant effect of *T. vulgaris* against NaNO_2_-induced hepatic dysfunction.

The elevated levels of ALT and AST, decreased total serum proteins, and the histopathological alterations that we observed, are all indicators of toxicity and also well-known quantitative measures of liver activity^39^. While functional liver damage can be indicated by high serum levels of AST, a more liver-specific indicator is the enzyme ALT. ALT catalyzes the conversion of alanine into pyruvate and glutamate, making serum levels of ALT a more reliable indicator of liver damage^40^. Our findings demonstrated that the membrane structure and integrity of the liver cells, which would otherwise have been destroyed by NaNO_2_, remained undamaged and uncongested, providing strong evidence for the protective effect of *T. vulgaris* extract also described previously^17,41^.

Sharma et al.^42^ noted that hepatoprotective plants are characterized by significant levels of polyphenols and flavonoids, major compounds also found in *T. vulgaris*. Flavonoids promote the expression of enzymes involved in the production of glutamylcysteine synthetase and thioredoxin, leading to increased intracellular GSH levels^43,44^.The antioxidant assays used in this study, which targeted five different types of ROS molecules (MDA, SOD, GSH, GSSG, and GSH/GSSG), confirmed the strong antioxidant effect of the *T. vulgaris* extract. Our findings are in agreement with those of previous studies that reported the high phenolic content and strong antioxidative effect of *T. vulgaris* extract^17^. Inhibition and decreased function of the antioxidant system causes the accumulation of H_2_O_2_ and hepatic cell decomposition^45^. Treatment with *T. vulgaris* extract significantly reversed these decreases in antioxidant enzyme activity.

Our study showed that NaNO_2_ intoxication reduced antioxidant activity and increased both oxidative stress and lipid peroxidation in agreement with previous reports^11,30^. Antioxidant defenses are affected by the expression of genes such as thioredoxin, SOD, and GSH^46^, and oxidative stress is regulated by several other downstream genes, including HO-1^47^. Hyun^48^ showed that many plant extracts play a critical role in increasing antioxidant activity and reducing ROS. Overexpression of a particular hemoxygenase-1 reported here in thymus control group and downregulation in the NaNO_2_-treated groups, confirmed the potential of *T. vulgaris* extract. HO-1 is normally involved in the regulation of mitochondrial biogenesis, neurogenesis, and angiogenesis^48^, causing an increase in protein expression^49^. As such, HO-1 plays an important role in mechanisms of oxidative stress and inflammation^50^. By controlling IL-1β activation^51^, HO-1 regulated the expression of inflammasome-associated genes^52^. The antioxidant mechanism is regulated by downstream genes that control oxidative stress, such as Nrf2 and HO-1^47,53^. Our findings confirmed that HO-1 expression plays a key role in the regulation of hepatic oxidative stress regulated by *T. vulgaris* extract. To explore the potential mechanism underlying the hepatoprotective effect of *T. vulgaris* extract, we examined the role of HO-1 signaling. Most reports have indicated that HO-1 plays a role in the activation and expression of some antioxidant and anti-inflammatory cytokine genes^54,55^. All were coincided with our reported findings.

Our study confirmed that *T. vulgaris* extract regulated the expression of pro-inflammatory cytokines, such as IL-1β, thereby ameliorating the NaNO_2_-induced inflammatory response. The inflammatory response causes increased production of IL-1β, IL-6, and TNF-α cytokines^56^, which play a leading role in sepsis and fever. NaNO_2_ intoxication has the same effect^57^, yet our study found that cytokine levels in mice pretreated with *T. vulgaris* extract returned to normal. Therefore, *T. vulgaris* extract prevented NaNO_2_-induced hepatic toxicity by ameliorating disrupted serum levels of IL-1β, IL-6, and TNF-α.

Reactive oxygen species (ROS) play a vital role in maintaining various human physiological processes^58^, but at high levels, they can be cytotoxic and cause liver damage^59^. Therefore, mechanisms exist to maintain appropriate ROS levels in the liver and other organs, thereby preventing oxidative stress and promoting redox homeostasis^59^. Our study found that while NaNO_2_ intoxication induced lipid peroxidation, pretreatment with *T. vulgaris* reversed these effects. In part, this was due to the recovery of depleted GSH, which plays a significant defensive role against oxidative stress^60^.

Hydroperoxides are converted into alcohols and water in a process catalyzed by thioredoxin, a cytosolic protein^61^. Thioredoxin has several antioxidant functions, including scavenging for free radicals, removing hydrogen peroxide, protecting against oxidative stress, as well as broader functions in genetic transcription, DNA and protein repair, immunostimulant roles, apoptosis, and cell proliferation. As a thiol-specific antioxidant, thioredoxin shares many of the same functions as glutathione systems and the two are understood to act in tandem in the management of oxidative stress^62^. In our study, NaNO_2_ downregulated thioredoxin and GSH synthase expression, but these returned to normal in mice pretreated with *T. vulgaris* extract.

In addition to biochemical alterations, the hepatotoxic effect of NaNO_2_ has been further implicated in a wide variety of degenerative, circulatory, and inflammatory alterations. The etiopathogenesis of these hepatopathic morphological alterations are likely to be multifactorial and mediated by NaNO_2_-induced oxidative damage and inflammation due to the formation of ROS and lipid peroxidation. The latter is associated with lysosomal and mitochondrial membrane rupture and subsequent release of digestive proteases^63^ and activation of the apoptosis-related Bax and caspase-3 genes^64^.

**Figure 5:**
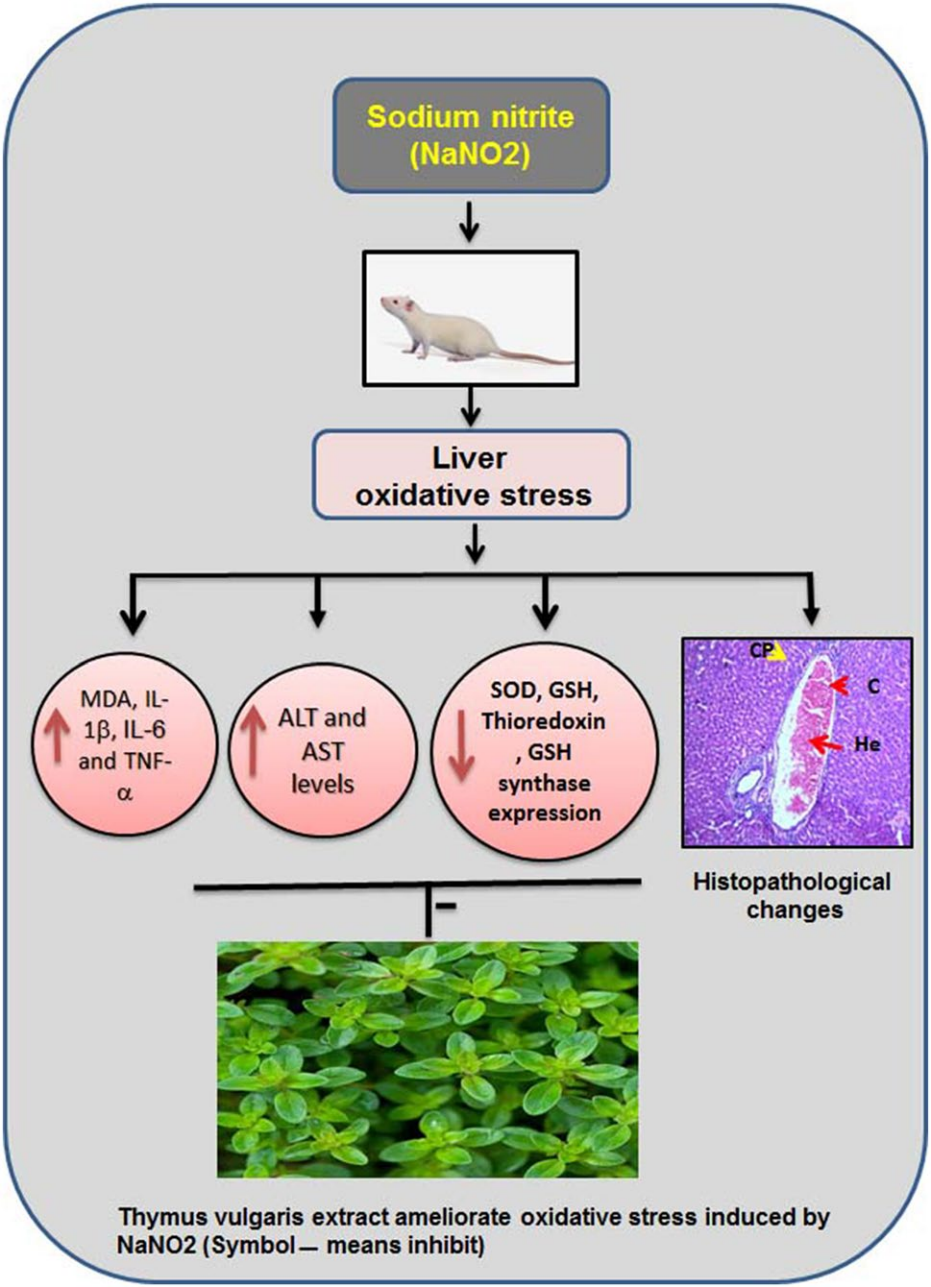
Schematic representation of the ameliorative effect of *T. vulgaris* extract on liver dysfunction. NaNO_2_ intoxication induced hepatic dysfunction, decreased antioxidant activity, and an increase in the levels and expression of inflammatory cytokines. These results of treatment with NaNO_2_ were normalized by pretreatment with *T. vulgaris* extract.

Treatment with NaNO_2_ caused hepatic dysfunction, oxidative stress, and alteration in the levels of inflammatory cytokines. Pretreatment with *T. vulgaris* extract alleviated these responses to toxicity and restored the levels and mRNA expression of the proteins and genes. The collective summary for the protective impact of *T. vulgaris* on NaNO_2_-induced liver dysfunction are clearly shown in Fig. 5.

## Conclusion

*T. vulgaris* extract exerted a protective effect against oxidative stress in liver tissues caused by NaNO_2_ intoxication. The effect was observed in different antioxidant systems and through the regulation of pro-inflammatory cytokines. In short, the common herb thyme (*T. vulgaris*) can be used in the prevention and treatment of hepatic oxidative stress.

## Data Availability

Data are available upon request.
